# Mediterranean Journal of Rheumatology June 2017 Highlights

**DOI:** 10.31138/mjr.28.2.62

**Published:** 2017-06-27

**Authors:** Dimitrios P. Bogdanos

**Affiliations:** Department of Rheumatology and Clinical Immunology, Faculty of Medicine, School of Health Sciences, University of Thessaly, Larissa, Greece

This issue of the Journal contains papers of great interest for the Reader. These highlights attempt to provide an overview of the papers and to quickly introduce them to our audience. The Reader will go through papers written by authors from various countries, including Greece, India, Iran, Iraq, Israel, Malta, and Turkey. The international reputation of the Journal increases, its visibility accelerates, attracting papers of high calibre considerably contributing to the field of rheumatology and the affiliated specialties.

Prof. Yehuda Shoenfeld’s review paper is an update on the current understanding of what he firstly described as ASIA (Autoimmune/inflammatory Syndrome Induced by Adjuvants) and the rest of us usually call Shoenfeld’s syndrome. ^1^ The review paper adds to the current knowledge about the syndrome. The updated major and minor diagnostic criteria of the syndrome are presented. We also learn that currently more than 4000 documented cases suffering from ASIA have been reported, making it not as rare as considered before. More importantly, the Authors underline basic concepts, which must be known by physicians to facilitate early and prompt diagnosis of the syndrome.

Prof. Aysen Akinci and Dr. Gamze Kilic review the available data assessing rehabilitation interventions for rheumatic diseases in the Mediterranean region. ^2^ As the Authors underline, at present, the rehabilitation programs should be designed as patient-centered using a multi-disciplinary approach and individual goals of therapy should also be clearly defined before the rehabilitation intervention. New data emerge suggesting that rehabilitation of specific patient groups may be more beneficial in warm climate settings such as the Mediterranean region, providing the rationale for better designing of studies in these countries. In another review paper, Prof. Sakkas provides an overview of the current knowledge of role of regulatory B cells, a relatively new subset of suppressor cells, in autoimmune rheumatic diseases. ^3^ The Author’s contribution to the field has been decisive, as his group was the first to describe the functional impairment of regulatory B cells in systemic sclerosis and more recently in psoriatic arthritis patients. From the overview, it appears that these cells not only play a role in the pathogenesis of these diseases, but also their state at baseline influences’ response to treatment and progression of the diseases over time. Tabrizi et al. ^4^ investigated the expression levels of microRNA expression profiles of the Drosha, Dicer and DGCR8 maturing microprocessor components in patients with ankylosing spondylitis. Recent data suggest that various microRNAs could be involved in the pathogenesis of spondylarthtitides including ankylosing spondylitis. Since Drosha, Dicer and DGCR8 are major microRNA biogenesis components, the Authors hypothesized that they could be involved in disease pathogenesis. Though their data do not show correlation between expression levels of the microRNAs with disease activity, they are suggestive of dysregulated expression levels which requires further investigation.

Grech and collaborators ^5^ investigated the manifestations of peripheral arterial disease in 100 patients with rheumatoid arthritis. The investigators noted that the majority of the patients had normal ankle brachial tibial artery index; however, waveform analysis was biphasic (abnormal) in approximately one-third of those. This finding has prompted them to go one step further suggesting that current recommendations about physiological testing of peripheral perfusion in rheumatoid arthritis should consider including waveforms as part of the assessment.

Dr. Trontzas, the ex-president of the Greek Rheumatology Society and Professional Association of Rheumatologists, position paper tackles the difficulties facing the rheumatologists practising in Greece, especially during the last few years of the extended monetary crisis. ^6^ The Author’s goal is to emphasize the urgent need for the Greek rheumatological community to embrace the new perception for the economic evaluation of medical procedures, to consent to the gradual change from the medical-oriented to a society-oriented model, to synergize efforts with state authorities, and finally, to organize a health system based on actual needs and social priorities.

The paper by Ravindran et al. ^7^ is addressing the challenges that journals of Rheumatology are currently facing. The Authors introduce the Indian Journal of Rheumatology, the official Journal of Indian Rheumatology Association and the challenges the Editors and the members of the Editorial Board face to increase the visibility of the Journal. Commonalities and differences amongst their Journal and ours are highlighted.

Prompted from an individual case is presenting, Dr. Ali Younis is reviewing the literature regarding the Crowned dens syndrome (CDS), a rare clinical entity characterized by acute neck pain due to calcification around the odontoid process of the axis in “crown-like” configuration. ^8^ The Author underlines the need for CDS to be considered in the differential diagnosis of acute neck pain, particularly in older patients and provide informative information regarding the clinical features of the syndrome, and the need for CT to assist accurate diagnosis which can be helpful in order to prevent misdiagnosis and unnecessary diagnostic procedures such as lumbar puncture and biopsy, as well as inappropriate treatment with antibiotics or antiviral drugs.

In their historical paper, Profs. Tsoucalas and Sgantzos reveal the decisive role that three Greek Byzantine physicians, ‘unknown’ to most of us, have played in the prompt management of gout. ^9^ The Authors report on the history of Severus Iatrosophista, Theophilus the Philosopher, and Jacobus Psychrestos, who were the first healers to use the plant Colchicum for the treatment of gout, long before others had implemented this plant in the management of podagra. According to the Authors, Rheumatology owes these three ‘forgotten’ Byzantine physicians an honorable citation, who still seek their fair place in the history of medicine.

We hope that the papers will be of interest for the Reader and we are committed to reiterate our focus on stimulating papers in our next issue.

Enjoy the reading.

## References

[1] Watad A Sharif K Shoenfeld Y The ASIA syndrome: basic concepts. Mediterr J Rheumatol 2017;28:64–9. 10.31138/mjr.28.2.64 PMC7046028 32185259

[2] Akinci A Kiliç G Future of Rehabilitation Interventions for Rheumatic Patients in the Mediterranean Region. Mediterr J Rheumatol 2017;28:70–4. 10.31138/mjr.28.2.70 PMC7046026 32185260

[3] Sakkas L I Regulatory B cells in autoimmune rheumatic diseases. Mediterr J Rheumatol 2017;28:75–9. 10.31138/mjr.28.2.75 PMC7046031 32185261

[4] Tabrizi Z Mansouri R Aslani A Jamshidi A R Mahmoudi M Expression levels of the microRNA maturing microprocessor complex components; Drosha, Dicer, and DGCR8 in PBMCs from ankylosing spondylitis patients. Mediterr J Rheumatol 2017;28:80–5. 10.31138/mjr.28.2.80 PMC7046025 32185262

[5] Grech C Gatt A A Borg A A Formosa C Determining the presence of Peripheral Arterial Disease in patients with Rheumatoid Arthritis. Mediterr J Rheumatol 2017;28:86–93. 10.31138/mjr.28.2.86 PMC7046034 32185263

[6] Trontzas P Rheumatology: Necessary adjustments to the realities of the new era in Greece. Mediterr J Rheumatol 2017;28(2):94–8. 10.31138/mjr.28.2.94 PMC7046029 32185264

[7] Ravindran V Misra D P Negi V S Challenges facing a Rheumatology journal: Role and credentials of editorial board members. Mediterr J Rheumatol 2017;28(2):99–100.

[8] Abdul-Rahman Younis A Crowned Dens Syndrome as a cause of acute neck pain: a Case Report and Review of the Literature. Mediterr J Rheumatol 2017;28(2):101–5. 10.31138/mjr.28.2.101 PMC7046027 32185265

[9] Tsoucalas G Sgantzos M Severus Iatrosophista, Theodosius the Philosopher and Jacobus Psychrestos, introducing Colchicum as an innovative treatment for podagra in the early Byzantine period. Mediterr J Rheumatol 2017;28(2):106–9. 10.31138/mjr.28.2.106 PMC7046033 32185266

